# Lung Transplantation for Pleuroparenchymal Fibroelastosis

**DOI:** 10.3390/jcm10050957

**Published:** 2021-03-01

**Authors:** Haruhiko Shiiya, Masaaki Sato

**Affiliations:** 1 Department of Thoracic Surgery, The University of Tokyo Graduate School of Medicine, 7-3-1 Hongo, Bunkyo-ku, Tokyo 113-8655, Japan; shiiyah-sur@h.u-tokyo.ac.jp; 2 Department of Cardiovascular and Thoracic Surgery, Hokkaido University Graduate School of Medicine, Kita 15 Nishi 7, Kita-ku, Sapporo 060-8638, Hokkaido, Japan

**Keywords:** lung transplantation, pleuroparenchymal fibroelastosis, interstitial lung disease, chest wall

## Abstract

Pleuroparenchymal fibroelastosis (PPFE), a new disease entity associated with interstitial pneumonia, is characterized by fibrosis and elastosis involving the pleura and subpleural lung parenchyma, predominantly in the upper lobe. As the awareness of this disease entity has increased, many studies have revealed the prevalence and incidence, clinical and pathological characteristics, and disease course of PPFE. Patients with PPFE reportedly have several unique clinical characteristics—including an extremely low body mass index with a slender body and chest wall deformity, known as “flat chest”. As this disease progresses, shrinking of the lungs often causes life-threatening complications, such as pneumothorax, and associated air leak syndrome. Lung transplantation is considered the only effective treatment for patients with advanced PPFE; however, little is known about the influences of the characteristics of PPFE on the outcome of lung transplantation. This review focuses on the unique clinicopathologic characteristics of PPFE and associated outcomes of lung transplantation for these patients.

## 1. Introduction

Pleuroparenchymal fibroelastosis (PPFE) is characterized by fibrosis involving the pleura and subpleural lung parenchyma, predominantly in the upper lobes. PPFE is broadly divided into two types: Idiopathic PPFE (IPPFE) and non-idiopathic PPFE. An associated condition termed idiopathic pulmonary upper lobe fibrosis was first reported by Amitani et al. [1] in 1992 in the Japanese literature. The term “idiopathic pleuroparenchymal fibroelastosis” was first used by Frankel et al. [2] in 2004, and IPPFE was formally included as a rare subtype in the international classification of the idiopathic interstitial pneumonias (IIPs) in 2013 [3]. Since then, the awareness of this disease entity has increased, and several characteristic clinical features have been reported; patients with IPPFE often have a slender body and a low body mass index (BMI) [4], and many develop a chest wall deformity known as “flat chest” [5]. In the advanced stage of the disease, shrinking of the lungs often causes pneumothorax and associated air leak syndrome (ALS) [6,7,8,9]. No medical treatment has been shown to be effective, and lung transplantation (LT) is, therefore, considered a therapeutic option for patients with advanced PPFE. Although few data regarding the outcomes of LT for patients with PPFE are available, complicated post-LT courses associated with the unique features of PPFE have been reported. This narrative review focuses on the difficulties, challenges, and future perspectives of LT for patients with PPFE.

## 2. Prevalence and Incidence of PPFE

The number of reports associated with PPFE has increased during the last decade. However, no international consensus regarding the diagnostic criteria has been established, and the accurate prevalence and incidence of PPFE, therefore, remain unknown. Before IPPFE was included in the new classification of IIPs in 2013, PPFE may have been diagnosed as other IIPs, mostly idiopathic pulmonary fibrosis (IPF). Most previous reports describing the prevalence and incidence of IPPFE were reviews of patients with IIPs. The reported prevalence of a PPFE pattern in patients with IIPs is around 5.8–10.4% [10,11,12,13]. Oda et al. [10] reported that 11 (10%) of 110 patients with IPF had radiological PPFE and that 9 (8.2%) of these patients’ conditions fulfilled the histologic criteria for PPFE. Nakatani et al. [11] retrospectively reviewed 205 patients who underwent surgical lung biopsy for interstitial lung diseases (ILDs) and found that 12 (5.8%) had PPFE. They also reported that the frequency of IPPFE among IIPs was 10.4% [11]. Shioya et al. [12] reviewed 375 patients with IIPs and found that 29 (7.7%) met radiological criteria for IPPFE. Lee et al. [13] reviewed 445 patients with IPF and identified PPFE in 28 (6.3%). The frequency of PPFE among patients with ILDs is reportedly higher when the analysis is limited to LT candidates. A Japanese single-institution study showed that among 118 patients with ILDs listed for LT, 30 (25%) were diagnosed with radiological PPFE [14]. We previously reported that 8 (28%) of 29 LT candidates with IIPs were diagnosed with IPPFE [15]. Many previous reports describing the prevalence of IPPFE were from Japanese authors. Whether the prevalence and incidence of IPPFE in the Japanese population are higher than those in Western populations should be investigated further.

Non-idiopathic PPFE reportedly occurs mainly after chemotherapy, allogeneic hematopoietic stem cell transplantation (HSCT), allogeneic bone marrow transplantation (BMT), and LT. Mariani et al. [16] reviewed high-resolution computed tomography examination findings from 700 HSCT recipients and 53 LT recipients. Among them, the imaging findings of two (0.28%) HSCT recipients and four (7.5%) LT recipients were identified as clinically and radiologically consistent with PPFE [16]. PPFE associated with connective tissue disease has also been reported. Enomoto et al. [17] reviewed 113 patients with connective tissue disease-related ILDs and found radiologic PPFE-like lesions in 21 (19%) patients. Recently, Bonifazi et al. [18] identified PPFE in 65 (18.1%) of 359 patients with systemic sclerosis. Most patients with restrictive allograft syndrome, one of the clinical subtypes of chronic lung allograft dysfunction (CLAD), reportedly show a histological PPFE pattern [19]. CLAD is reported to occur in approximately 50% of recipients by five years after LT [20], and restrictive allograft syndrome is reported to account for 25% to 35% of cases of CLAD [21].

## 3. Clinical Characteristics of PPFE

Many papers have described the characteristic clinical and laboratory findings of PPFE, and excellent review articles have been published [4,22,23]. In the present review, therefore, we focus on the characteristic features and complications that may affect the outcomes after LT.

### 3.1. BMI

In general, an underweight status before LT is reportedly associated with worse survival after LT, although the optimal cutoff value of the BMI varies among different reports. Upala et al. [24] reported a significant risk of mortality after LT in candidates with a BMI of <18.5 kg/m^2^ [relative risk, 1.36; 95% confidence interval (CI), 1.11–1.66]. Singer et al. [25] reported that a BMI of <18.5 kg/m^2^ was associated with a 35% increased mortality (95% CI, 10–66%). Fernandez et al. [26] reported that a BMI of <20 kg/m^2^ in a cohort of patients with restrictive lung disease was associated with an increased risk of mortality at one year after LT (odds ratio, 1.23; 95% CI, 1.02–1.48). Komatsu et al. [27] proposed an optimal BMI cutoff of <17.0 kg/m^2^ as indicative of a pre-LT underweight status.

Patients with IPPFE have been reported to have a lower BMI than patients with IPF [10,12,13,28]. Oda et al. [10] evaluated nine patients with PPFE and a usual interstitial pneumonia (UIP) pattern, and their BMI (mean ± standard deviation) was 18.6 ± 1.8 kg/m^2^. Shioya et al. [12] reported that the mean BMI of 29 patients with radiological IPPFE was 20.1 ± 3.25 kg/m^2^. Lee et al. [13] reported that the coexistence of radiological PPFE in patients with IPF was associated with a lower BMI (PPFE, 21.2 ± 3.0 kg/m^2^ vs. non-PPFE, 24.4 ± 3.1 kg/m^2^). Enomoto et al. [28] reported that 44 patients with radiological IPPFE had a median BMI of 17.2 kg/m^2^ [interquartile range (IQR), 14.7–18.5]. We previously reported that the median pre-LT BMI of Japanese recipients with IPPFE was 16.7 kg/m^2^ [29]. Among patients with connective tissue disease, a radiological PPFE-like region is reportedly associated with a low BMI (median, 19.8 kg/m^2^; IQR, 17.3–22.4) [17]. Tanizawa et al. [14] reviewed LT candidates with ILDs and reported that the median BMI of 30 patients with radiological PPFE was 15.9 kg/m^2^ (IQR, 14.8–17.2). Thus, a substantial proportion of patients with PPFE have an extremely low BMI, which might predict worse survival after LT.

No data regarding the association between the low BMI in patients with PPFE and post-LT outcomes are currently available. In general, severe malnutrition is considered to be a relative contraindication for LT [30]. Given that a substantial proportion of patients with PPFE has a low BMI, the post-LT outcomes in patients with PPFE might be poor; however, the low BMI may not necessarily represent malnutrition and lead to poor outcomes. Indeed, in our previous work, the overall survival after LT in patients with IPPFE was similar to that in patients with IPF [29]. In patients with cystic fibrosis, weight loss often occurs secondary to maldigestion, malabsorption, and associated malnutrition. Malnutrition in patients with cystic fibrosis is reportedly associated with worse survival and pulmonary function after LT [31,32]. Whether the low BMI in patients with PPFE results from malnutrition and affects post-LT outcomes should be investigated in further studies. Other parameters that reflect frailty or the nutritional status, such as the muscle mass [33], bioelectrical impedance analysis results [34], and nutritional risk index [35], should also be evaluated in future studies. Moreover, severe restrictive dysfunction in patients with PPFE might result in increased respiratory effort and energy consumption.

### 3.2. Flat Chest

**Figure 1:**
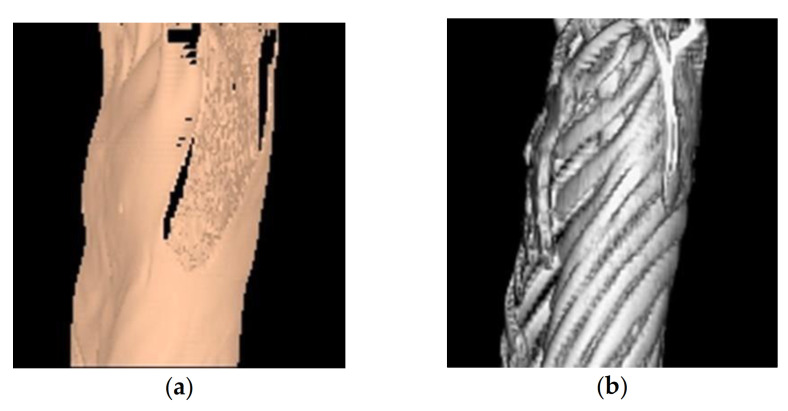
3D-CT of a patient with pleuroparenchymal fibroelastosis. (**a**) 3D-CT with body surface. Flattening of the chest wall is shown. (**b**) 3D-CT with bone reconstruction. The intercostal spaces are narrow, and the anteroposterior diameter is shortened. Abbreviation: 3D-CT—three-dimensional computed tomography.

Patients with PPFE often develop a flattening of the thoracic cage with the progression of the disease [5] (Figure 1). Chest deformity may result in the restriction of lung expansion after LT, and severe chest wall deformity is often considered an absolute contraindication for LT [30]; however, studies have shown that flat chest is not always associated with a poor outcome after LT. Miyoshi et al. [36] reported that pulmonary function after living-donor LT in patients with flat chest was poorer than that in patients with a normal chest, whereas the post-LT exercise capacity was equivalent, and the flat chest severity improved after LT. Miyahara et al. [37] reported that pulmonary function, exercise capacity, and survival in patients with flat chest after deceased-donor LT were similar to those in patients with a normal chest. Poorer pulmonary function after living-donor LT might be partly attributed to a lower volume of the donor lungs; living-donor LT usually uses two lung lobes, which have a smaller volume than the whole lungs used in deceased-donor LT. These two reports [36,37] were not limited to patients with PPFE; they included patients with other lung diseases and pulmonary hypertension. Patients with advanced ILDs and some congenital conditions, such as pectus excavatum, may show a flattening of the chest wall. Flat chest in patients with PPFE may be slightly different from that in patients with other diseases. In our previous report, pulmonary functions in patients with IPPFE after both deceased-donor and living-donor LT were poorer than those in patients with IPF [29]. These differences between patients with IPPFE and those with other diseases suggest that not only the lung volume itself, but other problems, such as a rigid chest wall, might persist and limit the function of patients with IPPFE even after LT. Yanagiya et al. [38] described a patient who required intensive pulmonary rehabilitation because of chest wall rigidity despite the fact that the chest wall flatness was reversed after LT. The mechanism underlying the development of flat chest in patients with PPFE should be investigated further.

### 3.3. Pneumothorax and ALS

Pneumothorax is one of the major complications in patients with both IPPFE and non-idiopathic PPFE. The incidence of pneumothorax in patients with PPFE is apparently higher than that in other IIPs. Enomoto et al. [28] reported that 8 (18%) of 44 patients with PPFE had a history of pneumothorax at the time of diagnosis. Lee et al. [13] reported that 5 (17.9%) of 28 patients who had IPF with radiological PPFE developed pneumothorax during the median follow-up period of 43.0 months. The incidence of pneumothorax is reportedly higher in LT candidates, who usually have advanced disease. Tanizawa et al. [14] reported that 24 (80%) of 30 patients with radiological PPFE had a history of pneumothorax at the time of registration for LT. We previously reported 31 recipients with IPPFE who underwent LT; among them, 16 (52%) had a history of pneumothorax at the time of LT [29].

**Figure 2:**
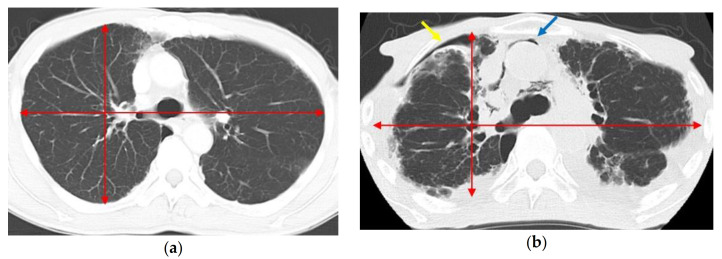
Chest CT images of a patient with idiopathic pleuroparenchymal fibroelastosis (IPPFE). (**a**) Chest CT before the diagnosis of IPPFE at the level of the sixth thoracic vertebra. The bilateral lungs are almost normal. (**b**) Chest CT 7 years later at the same level. The anteroposterior diameter of the thoracic cage has become shortened. Shrinking of the lungs has resulted in an air space in the right chest cavity (yellow arrow) and pneumomediastinum (blue arrow), indicating air leak syndrome. Abbreviations: CT—computed tomography; IPPFE—idiopathic pleuroparenchymal fibroelastosis.

Post-transplant ALS, including pneumothorax, subcutaneous emphysema, and pneumomediastinum, is known to occur after allogeneic HSCT or BMT [6,7,8,9]. PPFE-related ALS was recently recognized as a lung complication after allogeneic HSCT and allogeneic BMT [9,39]. Patients with IPPFE can also develop ALS at an advanced stage of the disease (Figure 2). ALS is sometimes life-threatening [6,7], can be recurrent [40], and often leads to fungal infection [6,41], which may result in mortality before LT.

Recurrent or prolonged pneumothorax and ALS sometimes necessitate interventions, such as pleurodesis and surgical repair, including pleural covering [9,42]. Severe adhesion in the chest cavity may lead to prolonged surgical and ischemic times and a higher rate of bleeding, resulting in delayed recovery and poorer outcomes after LT. These conditions may be exacerbated by longer cardiopulmonary bypass times. Drainage or observation may be preferred to pleurodesis in sufficient cases; however, pneumothorax in a future recipient for LT should be given the best immediate management to avoid missing the opportunity to undergo LT [30]. Pleurodesis and previous surgery are usually not contraindications for LT. Recurrent pneumothorax and ALS may cause adhesion even if pleurodesis is avoided.

## 4. Optimal Timing of LT

The natural course and prognosis of PPFE are highly variable. An analysis of 85 patients, including patients with both IPPFE and secondary PPFE, demonstrated a relatively long median survival of 11 years [22]. A subsequent study, however, revealed the presence of a rapidly progressing phenotype of IPPFE [43], and several studies have shown worse survival of patients with IPPFE than IPF [12,44]. The differences among these studies may be partly due to the timing of the diagnosis; most patients with IPPFE are diagnosed at the advanced stage. Patients with IPPFE may have a long subclinical stage with only small changes in the upper lobes [22]. Another important matter is the heterogeneity of PPFE. The coexistence of the PPFE pattern and other ILDs is apparently not uncommon [17,45]. Several studies have shown that the coexistence of a UIP pattern predicts a poorer prognosis in patients with IPPFE [13,46,47], although one study failed to show a significant impact of the presence of a UIP pattern on the prognosis of patients with IPPFE [28]. The coexistence of a PPFE pattern in patients with chronic hypersensitivity pneumonitis has also been reported to be associated with increased mortality [48]. In contrast, one study showed that after adjustment for age, sex, percent predicted forced vital capacity (FVC), and 6-min walk distance, patients with IPPFE and late-onset noninfectious pulmonary complications after HSCT and/or chemotherapy with a radiological PPFE pattern had better survival than patients with fibrotic ILDs without a radiological PPFE pattern [14]. Patients with IPPFE often show a disproportionate reduction in the FVC with a relatively preserved diffusing capacity [22]; therefore, the FVC may not always precisely represent the general condition in patients with IPPFE. Our previous small study of LT candidates suggested long-term survival despite unfavorable prognostic factors, including a low BMI and a short 6-min walk distance [15].

The optimal timing to list patients with PPFE on the waiting list for LT is still unknown. The proposal by the international guideline for the timing of listing patients with ILD for LT is based on a decline in the FVC, diffusing capacity, desaturation, and exercise capacity. The presence of pulmonary hypertension, pneumothorax, and acute exacerbation are also factors associated with the timing of listing [30]. Based on the current knowledge, the disease course of patients with PPFE is not always the same as that of patients with other ILDs, and some patients with PPFE may survive longer than patients with other ILDs; however, the survival of most patients with PPFE after diagnosis is as poor as that of patients with other ILDs.

## 5. Post-LT Course

Few data are available regarding the outcomes of LT for PPFE. Several authors have reported good post-LT courses without complications (Table 1). Chen et al. [49] described a patient with PPFE after chemotherapy for leukemia who was treated with single LT. The postoperative course was uneventful, and the patient was doing well four months after LT. Hata et al. [50] also reported an uneventful clinical course after living-donor lobar LT (LDLLT) for PPFE associated with chemotherapy. Rasciti et al. [51] described a patient with IPPFE who underwent bilateral LT. Although relapse of PPFE was suspected as a late complication, the postoperative course was uneventful. In contrast, several case reports have described complicated postoperative courses. Shimada et al. [52] reported PPFE associated with allogeneic umbilical cord stem cell transplantation. The patient underwent extracorporeal membrane oxygenation-bridged LDLLT and spent 30 days in the intensive care unit, but thereafter recovered and did well for one year. Yanagiya et al. [38] described a patient with IPPFE and flat chest who underwent LDLLT. The authors reported the necessity of intensive pulmonary rehabilitation for chest wall rigidity [38]. Righi et al. [53] also described a patient with IPPFE who required long-term rehabilitation after LT. We previously reported a case of fatal secondary pulmonary hypertension associated with flat chest in a patient with IPPFE who underwent single LT [54]. Other authors have also reported complicated post-LT courses [55,56,57]; however, after recovery from early complications, such as chylothorax, dysphagia, vocal cord paralysis, pneumonia, and repeated nausea and vomiting for an unknown reason, the patients apparently did well. There are also several case reports of LT for IPPFE or non-idiopathic PPFE without detailed information about the post-LT course [58,59,60]. To the best of our knowledge, our previous study of 31 patients with IPPFE in Japan is the largest study to show the outcomes after LT. In that study, although patients with IPPFE had a longer stay in the intensive care unit and the hospital, and although the low BMI and the low FVC persisted even after LT, the overall survival was similar to that of patients with IPF [29]. Based on the current knowledge, LT can be performed successfully and achieve acceptable survival for patients with PPFE; however, the post-LT course can be complicated, and some functional problems may persist even after LT. Further studies are necessary to obtain accurate knowledge regarding the outcome of LT for PPFE. The incidence of CLAD and other late complications should also be determined in further studies.

## 6. Conclusions

Few data are available regarding the optimal strategy to achieve the best outcome after LT for patients with PPFE. Further studies are needed to obtain accurate knowledge regarding LT for PPFE. Based on the current knowledge, unique characteristics of patients with PPFE may result in a complicated intraoperative and short-term post-LT course; however, the long-term outcomes are apparently similar to those of patients with other ILDs. As the only therapeutic option for patients with PPFE, LT should be considered in the advanced stage of the disease. Clinicians should consider the distinct characteristics of patients with PPFE when considering the possibility of LT, follow up patients on the waiting list, and follow up patients after LT. Further studies should also address the underlying mechanism of PPFE.

## Figures and Tables

**Table 1:** Published case reports describing lung transplantation for PPFE.

Author	Sex	Age	Underlying Condition	Procedure	Posttransplant Course	Outcome
Chen et al. (2014)	Male	14	Chemotherapy	Left single	uneventful	Alive
Portillo et al. (2015)	Male	25	Castleman’s disease	Bilateral	NA	Alive
Hata et al. (2016)	Male	19	Chemotherapy	LDLLT	uneventful	Alive
Yanagiya et al. (2016)	Female	27	Idiopathic	LDLLT	complicated	Alive
Huan et al. (2017)	Male	34	Idiopathic	Bilateral	NA	NA
Shimada et al. (2018)	Female	21	HSCT	LDLLT	complicated	Alive
Tsubosaka et al. (2018)	Male	19	Chemotherapy	Living-donor	NA	NA
Righi et al. (2018)	Male	42	Idiopathic	Bilateral	complicated	Alive
Ali et al. (2019)	Female	26	Idiopathic	Bilateral	complicated	Alive
Aljefri et al. (2019)	Male	27	Idiopathic	Bilateral	complicated	Alive
Sekine et al. (2020)	Female	29	Idiopathic	LDLLT	complicated	Alive
Rasciti et al. (2020)	Male	48	Idiopathic	Bilateral	possible PPFE relapse	Re-transplant
Shiiya et al. (2020)	Female	40	Idiopathic	Left single	complicated	Dead

Abbreviations: HSCT—hematopoietic stem cell transplantation; LDLLT—living-donor lobar lung transplantation; NA—not available; PPFE—pleuroparenchymal fibroelastosis.

## Data Availability

This study did not report any data.
